# Genetic Analysis and Species Specific Amplification of the Artemisinin Resistance-Associated Kelch Propeller Domain in *P*. *falciparum* and *P*. *vivax*

**DOI:** 10.1371/journal.pone.0136099

**Published:** 2015-08-20

**Authors:** Eldin Talundzic, Stella M. Chenet, Ira F. Goldman, Dhruviben S. Patel, Julia A. Nelson, Mateusz M. Plucinski, John W. Barnwell, Venkatachalam Udhayakumar

**Affiliations:** 1 Centers for Disease Control and Prevention, Center for Global Health, Division of Parasitic Diseases and Malaria, 1600 Clifton Rd, Mail Stop D-67, Atlanta, Georgia, United States of America; 2 Atlanta Research and Education Foundation/VA Medical Center, Decatur, Georgia, United States of America; 3 President’s Malaria Initiative, Atlanta, Georgia, United States of America; Centro de Pesquisa Rene Rachou/Fundação Oswaldo Cruz (Fiocruz-Minas), BRAZIL

## Abstract

*Plasmodium falciparum* resistance to artemisinin has emerged in the Greater Mekong Subregion and now poses a threat to malaria control and prevention. Recent work has identified mutations in the kelch propeller domain of the *P*. *falciparum* K13 gene to be associated artemisinin resistance as defined by delayed parasite clearance and *ex vivo* ring stage survival assays. Species specific primers for the two most prevalent human malaria species, *P*. *falciparum* and *P*. *vivax*, were designed and tested on multiple parasite isolates including human, rodent, and non- humans primate *Plasmodium* species. The new protocol described here using the species specific primers only amplified their respective species, *P*. *falciparum* and *P*. *vivax*, and did not cross react with any of the other human malaria *Plasmodium* species. We provide an improved species specific PCR and sequencing protocol that could be effectively used in areas where both *P*. *falciparum* and *P*. *vivax* are circulating. To design this improved protocol, the kelch gene was analyzed and compared among different species of *Plasmodium*. The kelch propeller domain was found to be highly conserved across the mammalian *Plasmodium* species.

## Introduction

Following the development and spread of resistance to antimalarials such as chloroquine and sulfadoxine-pyrimethamine, artemisinin-based combination therapy (ACT) was adopted as first-line treatment for uncomplicated *Plasmodium falciparum* malaria worldwide [1]. However, resistance to artemisinin, as measured by delayed parasite clearance, has now been confirmed in multiple countries in the Greater Mekong Subregion [2–10]. There is growing concern that artemisinin resistance may spread from this region to Africa and other parts of Asia as was the case with chloroquine and sulfadoxine-pyrimethamine resistance [11–13].

While *in vivo* therapeutic efficacy studies (TES) are considered the gold standard for determining anti-malarial efficacy, the WHO recommends that data from these studies be complemented with molecular markers of drug resistance [1]. After a long search to identify a specific locus implicated in artemisinin resistance, the kelch propeller domain of the K13 gene (*PF3D7_1343700*) on chromosome 13 was recently identified as a molecular marker of artemisinin resistance [14]. Several mutations in the kelch propeller domain have now been associated with *in vitro* ring stage survival assays and delayed parasite clearance rates in patients treated with artemisinins [8, 14]. As a result, sequencing the kelch propeller domain of the K13 gene is becoming an important tool in the global surveillance of antimalarial drug resistance in *P*. *falciparum* [8–10, 14–19].

While *P*. *falciparum* is the dominant species that causes human malaria worldwide and contributes the most to mortality, *P*. *vivax* is more prevalent than *P*. *falciparum* in many regions of the Greater Mekong Subregion [20]. Interestingly, a recent study showed that non-synonymous mutations in the *P*.*vivax* ortholog K12 gene are already circulating at very low frequencies in Cambodia [21]. While chloroquine remains the primary treatment option for *P*. *vivax* infections, ACTs have also been found to be efficacious in clinical trials in Asia and may be used as an alternative treatment to chloroquine [22, 23]. In addition, widespread use of ACTs for *P*. *falciparum* infection may exert some indirect selective pressure on the *P*. *vivax* kelch gene in individuals with mixed *P*. *falciparum* and *P*. *vivax* infections. Moreover, in areas where mefloquine has been extensively used as the first-line treatment in falciparum-uncomplicated malaria, high frequencies of *P*. *falciparum* and *P*. *vivax* isolates with increased *mdr-1* copy numbers [24] have been observed. These findings suggest that antimalarial drugs used to treat *falciparum* malaria may have a significant impact on sympatric *Plasmodium* species [21].

In areas where both *P*. *falciparum* and *P*. *vivax* coexist, kelch gene amplification must be species specific; otherwise, non-specific amplification using *P*. *falciparum* and *P*. *vivax* clinical isolates, might lead to incorrect classification of polymorphisms. Although microscopy remains standard practice for malaria diagnosis, molecular methods such as PCR based protocols for the confirmation of *Plasmodium species* can also be used [25]. Here, we provided a *P*. *falciparum* and a *P*.*vivax* species-specific protocol for accurately amplifying and sequencing the kelch propeller domain of these two species.

## Materials and Methods

### Parasite isolates

The following species and strains of parasites archived at the Malaria Laboratory, Research and Development Unit, Center for Disease Control and Prevention (CDC) were analyzed: *P*. *falciparum* (strains: W2, 7G8, FCR3, Dd2, 3D7, HB3, Honduras I/CDC, Panama II, Brazil, Malayan IV, and Santa Lucia), *P*. *vivax* (strains: India VII, Nicaragua I, Belem, Mauritania II, Ecuador I, Eritrea I, Indonesia XIX, and Vietnam II), *P*. *malariae* (Uganda I), *P*. *ovale* (Nigeria I), *P*. *reichenowi*, *P*. *cynomolgi* (strains: PTI, Berok, Cambodia, Smithsonian, Gombok), *P*. *fieldi* (AB introlatus), *P*. *simiovale*, *P*. *simium*, *P*. *knowlesi* (strains: Philippines, Malayan), *P*. *gonderi*, *P*. *hylobati*, *P*. *inui* (Taiwan, Philippines), *P*. *berghei*, *and P*. *yoeli*. DNA was isolated using the commercially available QIAamp DNA Mini Kit (QIAGEN, Valencia, CA, USA). Genomic DNA was eluted with 100 μl of elution buffer and stored at −20°C for use in PCR assays. All isolates were screened using PET-PCR [26, 27] and confirmed to be positive for *Plasmodium*.

### Species specific primer design and genetic analysis

Primers specific for the kelch K13 gene (PF3D7_1343700) for *Plasmodium falciparum* and the ortholog kelch K12 gene in *Plasmodium vivax* were designed using the Geneious Pro R8 software (www.geneious.com). Briefly, Geneious Pro R8 was used to perform a BLAST search using the reference *P*. *falciparum* K13 gene sequence (PF3D7_134700) and download matching and published kelch gene *Plasmodium* sequences (Accession number: *NC_009917*, *P*.*vivax; NC_020405*, *P*. *cynomolgi strain B; NW_008481840P*. *inui; NC_011913*, *P*. *knowlesi; NW_672329*, *P*. *berghei; NC_020405*, NW_875089; *P*.*chabaudi;* and NW_865350, *P*. *yoelii; CDO66221*, *protein*, *P*. *reichenowi)*. Sequences were aligned and analyzed for regions of sequence identity. Using the principle of allelic exclusion, different sets of species specific primers were designed and mapped onto the aligned sequences, aiming for primers that differentiate species by destabilizing the 3’ end via mismatched nucleotides. We tested *P*. *falciparum* and *P*. *viax* species specific primers on various *P*. *falciparum* and *P*. *vivax* strains derived from various geographical origins as well as on other human, non-human primate, and rodent malaria species. Lastly, our protocol was compared to an original published protocol for the amplification of the K13 gene in *P*. *falciparum* [14].

### PCR amplification and sequencing of the *Plasmodium* kelch propellor domain

The kelch gene from different *Plasmodium* species was amplified using a nested PCR approach. The species specific primers used for amplification of the kelch propeller domain are shown in Table 1. Two μL of genomic DNA was amplified using 0.5 μM of each primer, 0.2mM dNTP, 2 mM MgCl_2_ for the primary and secondary reactions, and 1 U Expand High Fidelity Taq (Roche CA, USA). For the primary reaction the following cycling parameters were used: 5 min at 94°C, 35 cycles at 94°C for 30 sec, 46°C for 60 sec (for *P*. *falciparum* primers) or 61°C for 60 sec (for *P*. *vivax* primers), 72°C for 90 sec, and final extension for 5 min at 72°C. For the nested PCR, 1μL of 1/5 diluted primary PCR product was used as template. For the nested PCR reaction the following cycling parameters were used: 2 min at 94°C, 35 cycles at 94°C for 30 sec, 55°C for 30 sec (*P*. *falciparum* primers) or 59°C for 30 sec (*P*. *vivax* primers), 72°C for 90 sec, and final extension for 5 min at 72°C. PCR products were confirmed using a 2% agarose gel electrophoresis and Gel red (Biotium, Hayward, CA USA). For cycle sequencing, 2.0uL Big Dye and Dye Buffer, 0.32 uL Primer (stock primer concentration of 10uM), 1.0uL of 1/5 diluted secondary PCR product, and 5.32uL of sterile water were used per reaction with the following cycling parameters: 96.0°C for 60 sec, 30 cycles 96.0°C for 10s, 50.0°C for 5 sec, and 70.0°C for 4 min. The original K13 amplification protocol [14] was used for comparison with the protocol described in this study. Sanger sequencing of PCR products was performed using ABI 3130 (Applied Biosystems, CA, USA). The sequence data was analyzed using Geneious Pro R8 software (www.geneious.com).

**Table 1:** *P*. *falciparum* and *P*. *vivax* K13 propeller domain primers. Primary and secondary PCR primers for each species and respective annealing temperatures are shown.

Species	Primer Name	Primer	Annealing Temperature	PCR
*P*. *falciparum*	K13_Pf_F1	5’-GCAAATAGTATCTCGAAT-3’	46°C	1° reaction
	K13_Pf_R1	5’-CTGGGAACTAA TAAAGAT-3’		
	K13_Pf_F2	5’-GATAAACAAGGAAGAATATTCT-3’	54°C	2° reaction
	K13_Pf_R2	5’-CGGAATCTAATATGTTATGTTCA-3’		
*P*. *vivax*	K13_Pvx_F1	5’-CATTTCCAACTTCTCCGTC-3’	61°C	1° reaction
	K13_Pvx_R1	5’-TATCTGCCACTCATTCGTG-3’		
	K13_Pvx_F2	5’-CGAAAGTGAGGCTTTACTA-3’	59°C	2° reaction
	K13_Pvx_R2	5’-CCACCAGTGATGATGTAC -3’		

### *Plasmodium* kelch propeller sequences submitted to Genbank

All of the geographically distinct *P*. *falciparum* and *P*. *vivax* isolates sequenced for the propeller domain were identical on both the nucleotide and amino acid level to the *P*. *falciparum* 3D7 *(PF3D7_134700)* isolate and *P*. *vivax* Sal-1 (*NC_009917)* isolate already available in Genbank, respectively. The following non-human primate isolates were also successfully amplified and sequenced: *P*. *cynomolgi (Gombok)*, *P*. *simium*, *P*. *simiovale* and *P*. *inui*. Any new sequences reported in this study were deposited in Genbank under the accession numbers: KT198970-KT198972.

### 3D structural modeling of the loop region

The protein structure of the Keap1 protein (Protein Data Bank: 2Z32) was used to generate the *P*. *falciparum* K13 propellor domain using the USCSF Chimera plugin MODELLER [28].

## Results

### Comparison of the kelch propeller domain in human, non-human primates, and rodent *Plasmodium* species

**Fig 1:**
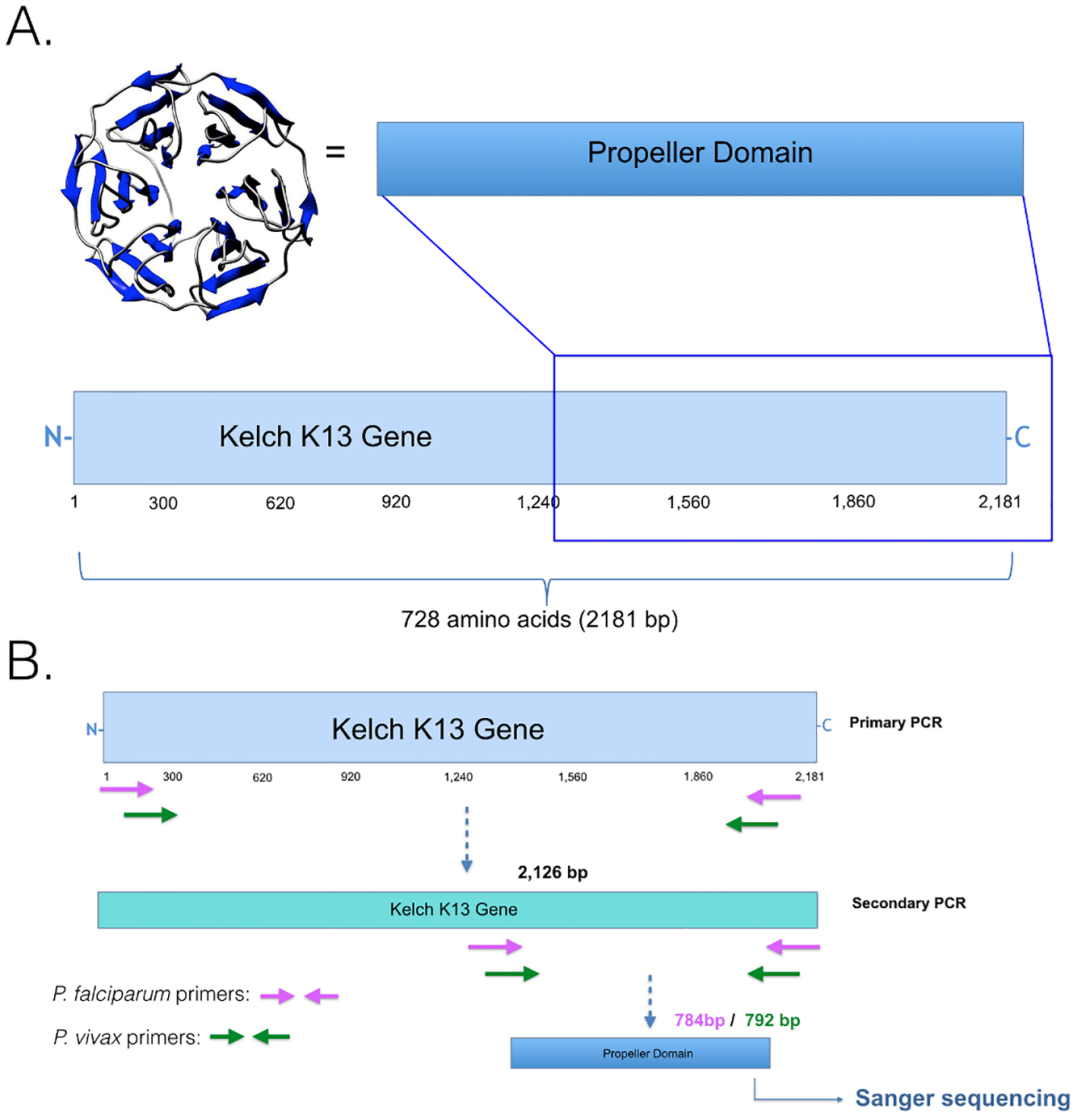
Schematic representation of the *P*. *falciparum* K13 gene. (A) Schematic depicting the *P*. *falciparum* K13 gene size and propeller domain region. A 3D model is shown for the predicted propeller domain. (B) Species specific nested PCR workflow for amplifying the K13 gene and propeller domain. The protein structure of the Keap1 protein (Protein Data Bank: 2Z32) was used to generate the 3D model of the *P*. *falciparum* K13 propellor domain.

**Fig 2:**
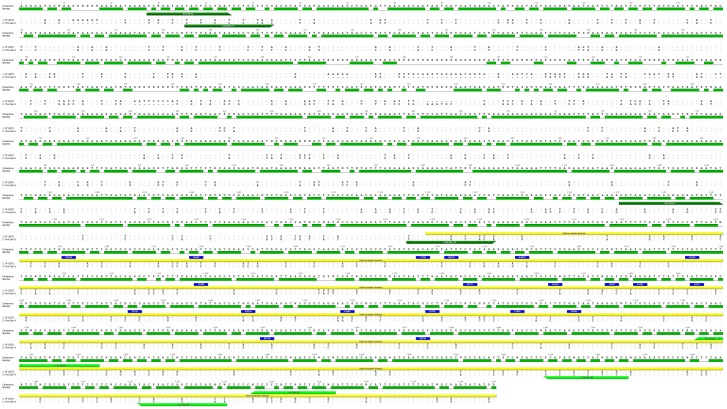
Alignment of kelch gene for *P*. *falciparum* and *P*. *vivax*. Nucleotide and amino acid sequence alignment of the kelch gene for *P*. *falciparum* and *P*. *vivax;* disagreements in nucleotide (black) and amino acid (color) are highlighted. The yellow annotation above the sequence indicates the gene region that encodes the kelch propeller domain; blue squares over the *P*. *falciparum* sequence denotes K13 propeller mutations reported by Ariey et al [14]. Forward primers are annotated in dark green and reverse primers in light green. Shown are sequences for the isolates 3D7 (*P*. *falciparum*) and Sal-1 (*P*. *vivax)*.

The *P*. *falciparum* (PF3D7_1343700) K13 gene is 2,181 nucleotide base pairs, encoding a protein of 727 amino acids (Fig 1), whereas the *P*. *vivax* (Sal-1 NC_009917) ortholog K12 gene is 2,139 base pairs, corresponding to 713 amino acids. The difference in length is due to nucleotide deletions found at the 5’ end of the *P*. *vivax* sequence at positions: 12, 407, 446, 472, and 534 with respect to the *P*. *falciparum* sequence (Fig 2). Comparison of the entire coding region of the kelch gene between *P*. *falciparum* and *P*. *vivax* revealed the gene to be 80% identical at the nucleotide level and 88% identical at the amino acid level. However, when the comparison was restricted to the kelch propeller domain only, the two genes were 80% identical at the nucleotide level and 97% at the amino acid level (Table 2).

**Table 2:** Genetic distance matrix of kelch propeller domain of various *Plasmodium* species. Amino acid and nucleotide sequence percent (%) identify (e.g. percent of residues that are identical) are shown in the table. Amino acid similarity is shown in bold numbers and nucleotide similarity in italicized numbers.

-	P.fal	P.rei	P.viv	P.cyn	P. cyn G	P.kno	P.inu	P.simi	P.sim	P. yoe	P. cha
P.fal	-	**100**	**97**	**97**	**97**	**97**	**97**	**97**	**96**	**95**	**95**
P.rei	*N/A*	-	**97**	**97**	**97**	**97**	**97**	**98**	**96**	**95**	**95**
P.viv	*80*	*N/A*	-	**100**	**100**	**99**	**100**	**100**	**99**	**96**	**96**
P.cyn	*82*	*N/A*	*95*	-	**100**	**99**	**100**	**100**	**99**	**96**	**96**
P. cyn G	*83*	*N/A*	*94*	*96*	-	**99**	**100**	**100**	**100**	**94**	**95**
P.kno	*82*	*N/A*	*92*	*94*	*93*	-	**99**	**99**	**98**	**98**	**95**
P.inu	*81*	*N/A*	*94*	*96*	*96*	*93*	-	**100**	**99**	**96**	**96**
P.simi	*83*	*N/A*	*95*	*97*	*96*	*94*	*97*	-	**100**	**95**	**95**
P.sim	*80*	*N/A*	*100*	*95*	*94*	*92*	*94*	*95*	-	**93**	**94**
P. yoe	*87*	*N/A*	*79*	*81*	*82*	*82*	*80*	*81*	*79*	-	**99**
P. cha	*86*	*N/A*	*80*	*81*	*82*	*82*	*80*	*81*	*80*	*97*	-

*P*.*fal* = *P*. *falciparum; P*.*rei = P*.*reichenowi; P*.*viv = P*.*vivax; P*.*cyn = P*.*cynomolgi; P*.*cyn G = P*. *cynomolgi Gombok; P*.*kno = P*.*knowlsi; P*.*inu = P*. *inui; P*. *simi = P*. *simiovale; P*. *sim = P*. *simium; P*.*yoe = P*. *yoelii; P*.*cha = P*.*chabaudi;* N/A = not available.

All of the geographically distinct *P*. *falciparum* and the *P*. *vivax* isolates sequenced for the kelch propeller domain were identical on both the nucleotide and amino acid level to the *P*. *falciparum 3D7* (PF3D7_134700) isolate and *P*. *vivax Sal-1* isolate (NC_009917), respectively. A total of eight amino acid differences within the kelch propeller domain (spanning 301 amino acids in length) between all of the *P*. *falciparum* and of *P*. *vivax* strains were found at the following positions: 448, 517, 519, 568, 578, 605, 691, and 708 (Table 3). The kelch propeller domain is relatively conserved among the rodent and primate species of *Plasmodium* (Table 2, S1 and S2 Figs). Nucleotide and amino acid comparison for the various *Plasmodium* species is presented in Table 2.

**Table 3:** *P*. *falciparum* non-synonymous amino acid changes in the kelch propeller domain as compared to *P*. *vivax*. Relative to alignment between *P*. *falciparum* 3D7 and *P*. *vivax* Sal-I.

	*P*. *falciparum*				*P*. *vivax*			
Codon Position	Amino Acid	Nucleotide	Side-Chain Polarity	Side-Chain Charge	Amino Acid	Nucleotide	Side-Chain Polarity	Side-Chain Charge
448	**I**	ATA	Nonpolar	Neutral	**M**	ATG	Nonpolar	Neutral
517	**V**	GTA	Nonpolar	Neutral	**T**	ACT	Polar	Neutral
519	**Y**	TAT	Polar	Neutral	**F**	TTT	Nonpolar	Neutral
568	**V**	GTG	Nonpolar	Neutral	**I**	ATC	Nonpolar	Neutral
578	**A**	GCT	Nonpolar	Neutral	**S**	TCC	Polar	Neutral
605	**E**	GAA	Polar	Negative	**D**	GAT	Polar	Negative
691	**E**	GAA	Polar	Negative	**D**	GAT	Polar	Negative
708	**L**	CTT	Nonpolar	Neutral	**I**	ACT	Nonpolar	Neutral

### Kelch propeller domain species specific amplification

**Fig 3:**
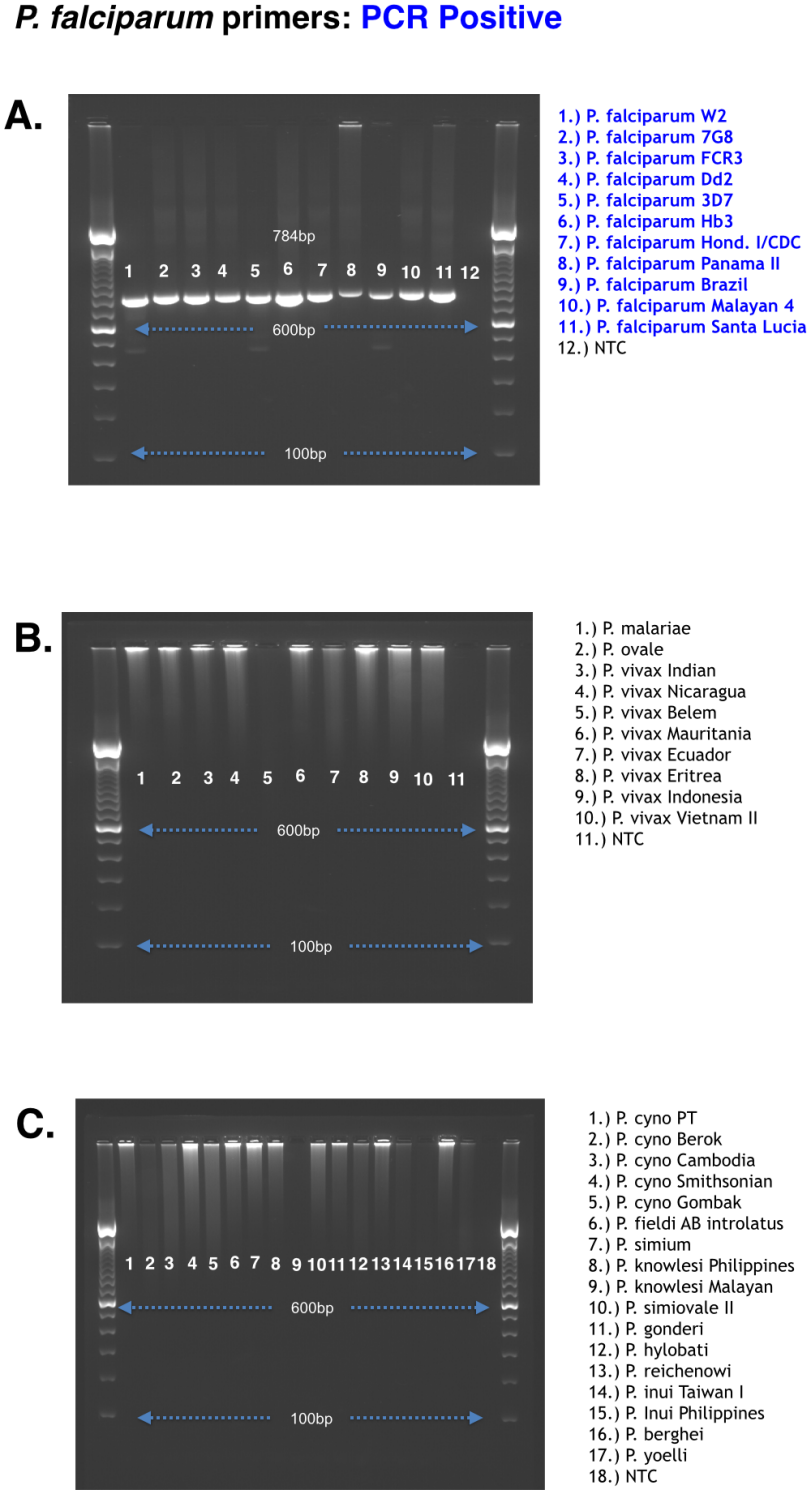
*P*. *falciparum* K13 gene species-specific amplification. The *Plasmodium falciparum* species specific primers were tested on multiple *Plasmodium* species, including human, rodent, and non-human primate malaria parasites from various geographical regions. The expected K13 propeller domain PCR product for *P*. *falciparum* is 784 base pairs. Panels (A) and (B) show human malaria parasites tested; Panel (C) shows non-human primate and rodent malaria parasites tested. Blue text denotes samples that were amplified by the PCR protocol.

The final *P*. *falciparum* nested PCR product size using species specific primers was 784 bp for *P*. *falciparum* and 792 bp for *P*. *vivax*. The *P*. *falciparum* primers designed in this study amplified all *P*. *falciparum* strains tested, but not any other *Plasmodium* species (Fig 3). In contrast, the previously published protocol showed non-specific amplification with other *Plasmodium* species, S3 Fig.

**Fig 4:**
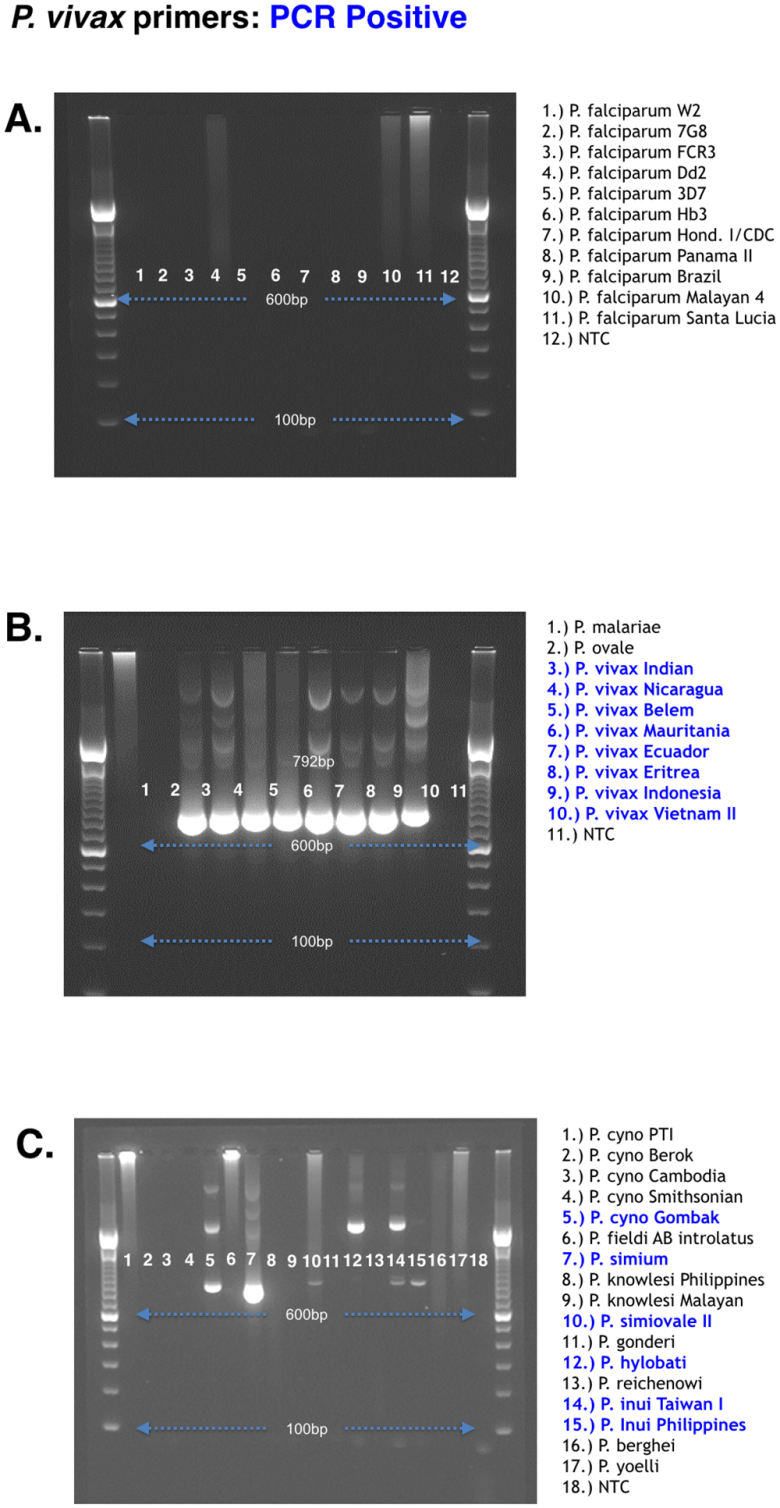
*P*. *vivax* K12 gene species-specific amplification. The *Plasmodium vivax* species specific primers were tested on multiple *Plasmodium* species, including human, rodent, and non-human primate malaria parasites from various geographical regions. The expected K12 propeller domain PCR product for *P*. *vivax* is 792 base pairs. Panels (A) and (B) show human malaria parasites tested; Panel (C) shows non-human primate and rodent malaria parasites tested. Blue text denotes samples that were amplified by the PCR protocol.

The *P*.*vivax* primers amplified eight different *P*. *vivax* strains derived from various geographical regions as well as five closely related non-human primate malaria parasites: *P*. *cynomolgi*-Gombok strain, *P*. *simium*, *P*. *simiovale*, *P*. *inui*, *and P*. *hylobati* (Fig 4). However, only *P*. *cynomolgi*-Gombok strain, *P*. *simium*, *P*. *simiovale*, and *P*. *inui* could be sequenced. Interestingly, of the five *P*. *cynomolgi* strains tested, only the Gombok strain showed cross reactivity with the *P*. *vivax* primers (Fig 4). Neither the *P*. *falciparum* nor *P*. *vivax* primers amplified the other human malaria parasites *P*. *malariae*, *P*. *ovale* and *P*. *knowlesi*, or the rodent malaria parasites *P*. *bergehi* and *P*. *yoelii* (Figs 3 and 4).

## Discussion

In some endemic regions often *P*. *vivax* is more prevalent than *P*. *falciparum* [29, 30], and co-infections can be common. Artemisinin combination therapy has been in use since the early 2000s in numerous malaria endemic regions where *P*. *falciparum* and *P*. *vivax* co-exist [1]. While drug resistance in *P*. *falciparum* has primarily been the recent focus of research, the study of resistance in *P*. *vivax* has received limited attention. For example, to date only one other paper investigating kelch propeller domain mutations in *P*. *vivax* has been published [21]. In this study, we successfully designed a species specific protocol for the detection of K13 propeller domain mutations associated with artemisinin drug resistance in *P*.*falciparum*

The previously published protocol [14] was found to show non-specific amplification with multiple *Plasmodium* species (S3 Fig), including the two major human malaria species *P*. *falciparum* and *P*. *vivax*. While we were unable to successfully sequence the full length *P*. *vivax* kelch propeller domain using the previous protocol, we did obtain truncated sequence data for *P*. *vivax* using the published *P*. *falciparum* primers [14]. This can cause some uncertainty in the interpretation of sequence data.

The *P*. *falciparum* primers in our study were able to amplify different strains of *P*. *falciparum* indicating that this protocol will be useful for amplify and sequence the kelch propeller domain from several *P*.*falciparum* isolates from various regions around the world (Brazil, Honduras, Panama, and Malaysia). Importantly, the *P*. *falciparum* primers did not show amplification of any other human *Plasmodium* parasites (Fig 3).

Similarly, the *P*. *vivax* primers did not cross react with *P*. *falciparum* or any other human malaria parasites. Further, the *P*. *vivax* primers amplified successfully *P*. *vivax* strains collected from different geographical origins (Fig 3). The *P*. *vivax* primers showed evidence of some cross-reaction with *P*. *cynomolgi*-Gombok strain, *P*. *simium*, *P*. *simiovale*, and *P*. *inui* (Fig 4). This was expected, since most of these species are evolutionarily and genetically related to *P*. *vivax* [31]. Out of the five *P*. *cynomolgi* lab isolates tested only the Gombok strain cross-reacted with the *P*. *vivax* primers, which could be attributed to the known diversity within the two major sub-groups of *P*. *cynomolgi* [31]. Although this primer set was cross reactive with some of the non-human primate malaria parasites, this may not be a limitation for the amplification of field samples since most of these non-human primate parasites are not commonly transmitted naturally to humans. The only currently known zoonotic malaria is caused by *P*. *knowlesi*, which showed no cross reaction with either the *P*. *falciparum* or *P*. *vivax* species specific protocol.

Given that currently the K13 gene can serve as a effective molecular marker of artemisinin resistance, we emphasize the importance of using our species specific protocol for routine screening of K13 artemisinin associated resistant alleles.

## Supporting Information

S1 Fig Nucleotide sequence alignment of the kelch propeller domain of various *Plasmodium* species. Sequences of the kelch propeller domain are shown for *P*. *falciparum*, *P*. *vivax*, *P*. *cynomolgi strain B*, *P*. *cynomolgi Gombok*, *P*. *knowlesi*, *P*. *inui*, *P*. *simiovale*, *P*. *simium*, *P*. *yoelii*, *and P*. *chabaudi*. Nucleotides are highlighted based on disagreement with the reference *P*. *falciparum* K13 sequence. The yellow annotation below the *P*. *falciparum* sequence indicates the gene region that encodes the kelch propeller domain.
(PDF)

S2 Fig Amino acid sequence alignment of the kelch propeller domain of various *Plasmodium* species. Translation of kelch propeller domain nucleotide sequences are shown for *P*. *falciparum*, *P*. *reichenowi*, *P*. *vivax*, *P*. *cynomolgi strain B*, *P*. *cynomolgi Gombok*, *P*. *knowlesi*, *P*. *inui*, *P*. *simiovale*, *P*. *simium*, *P*. *yoelii*, *and P*. *chabaudi*. Highlighted are amino acid bases that are in disagreement with the reference *P*. *falciparum* kelch propeller sequence. The yellow annotation below the *P*. *falciparum* sequence indicates the gene region that encodes the kelch propeller domain.
(PDF)

S3 Fig K13 gene amplification using a previously described protocol. The original K13 gene amplification protocol was tested on multiple *Plasmodium* species, including human, rodent, and non-human primate malaria parasites from various geographical regions. Rows (A) and (B) show human malaria parasites tested; Row (C) shows non-human primate and rodent malaria parasites tested. Results are shown separately for the primary and secondary reactions. Blue text denotes samples that were amplified by the PCR protocol.
(TIFF)
