# Supplementary material for: Genetic Analysis and Species Specific Amplification of the Artemisinin Resistance-Associated Kelch Propeller Domain in P. falciparum and P. vivax
Source: PLoS One. 2015 Aug 20;10(8):e0136099. doi: 10.1371/journal.pone.0136099 (PMC4546394; doi:10.1371/journal.pone.0136099)
Supplement: S1 Fig — Sequences of the kelch propeller domain are shown for P. falciparum, P. vivax, P. cynomolgi strain B, P. cynomolgi Gombok, P. knowlesi, P. inui, P. simiovale, P. simium, P. yoelii, and P. chabaudi. Nucleotides are highlighted based on disagreement with the reference P. falciparum K13 sequence. The yellow annotation below the P. falciparum sequence indicates the gene region that encodes the kelch propeller domain. (PDF) [file pone.0136099.s001.pdf]

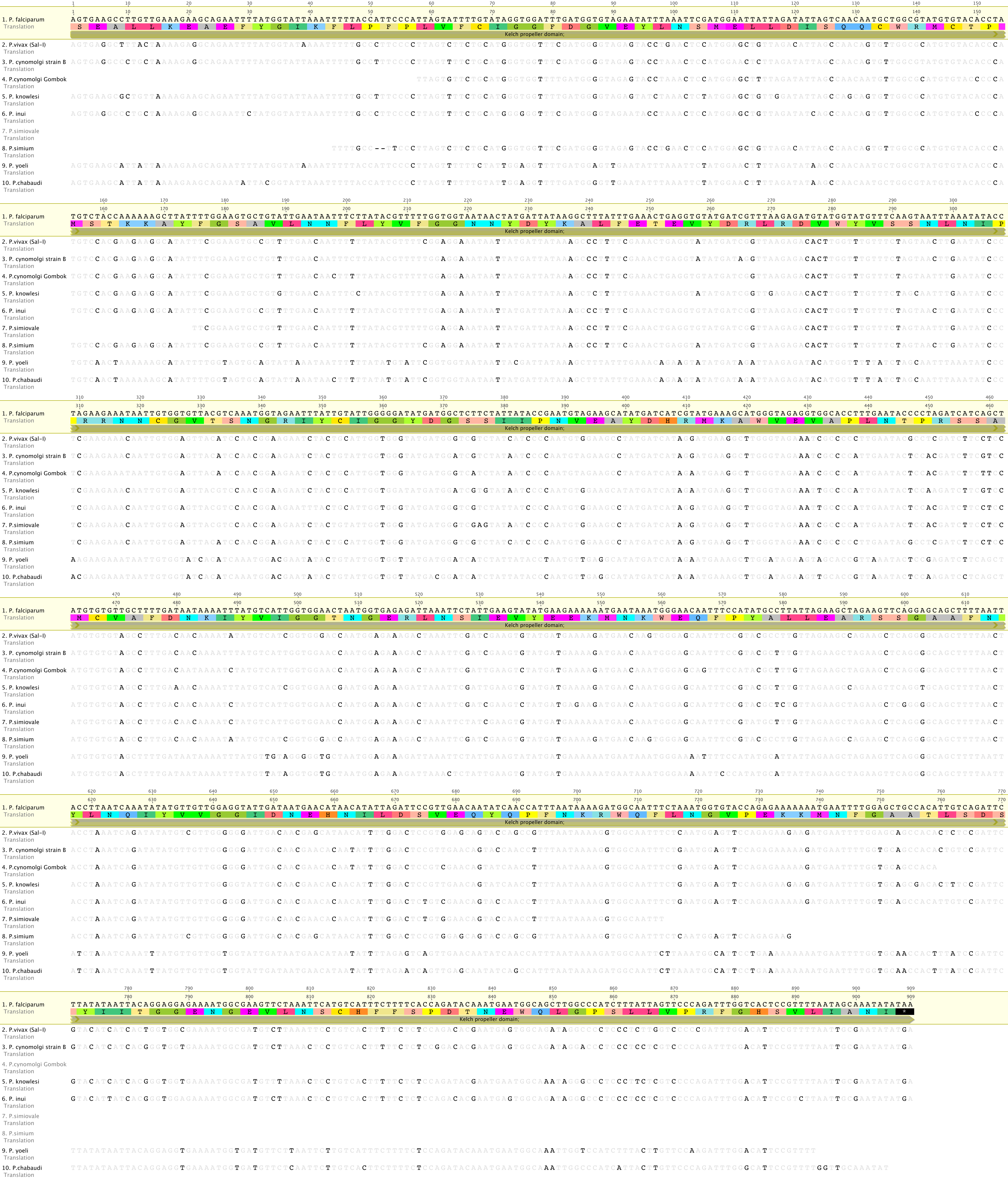
1. P. falciparum
Translation
Kelch propeller domain;
2. P.vivax (Sal-I)
3. P. cynomolgi strain B
4. P.cynomolgi Gombok
5. P. knowlesi
6. P. inui
7. P.simiovale
8. P.simium
9. P. yoeli
10. P.chabaudi
