# Supplementary material for: Genetic Analysis and Species Specific Amplification of the Artemisinin Resistance-Associated Kelch Propeller Domain in P. falciparum and P. vivax
Source: PLoS One. 2015 Aug 20;10(8):e0136099. doi: 10.1371/journal.pone.0136099 (PMC4546394; doi:10.1371/journal.pone.0136099)
Supplement: S2 Fig — Translation of kelch propeller domain nucleotide sequences are shown for P. falciparum, P. reichenowi, P. vivax, P. cynomolgi strain B, P. cynomolgi Gombok, P. knowlesi, P. inui, P. simiovale, P. simium, P. yoelii, and P. chabaudi. Highlighted are amino acid bases that are in disagreement with the reference P. falciparum kelch propeller sequence. The yellow annotation below the P. falciparum sequence indicates the gene region that encodes the kelch propeller domain. (PDF) [file pone.0136099.s002.pdf]

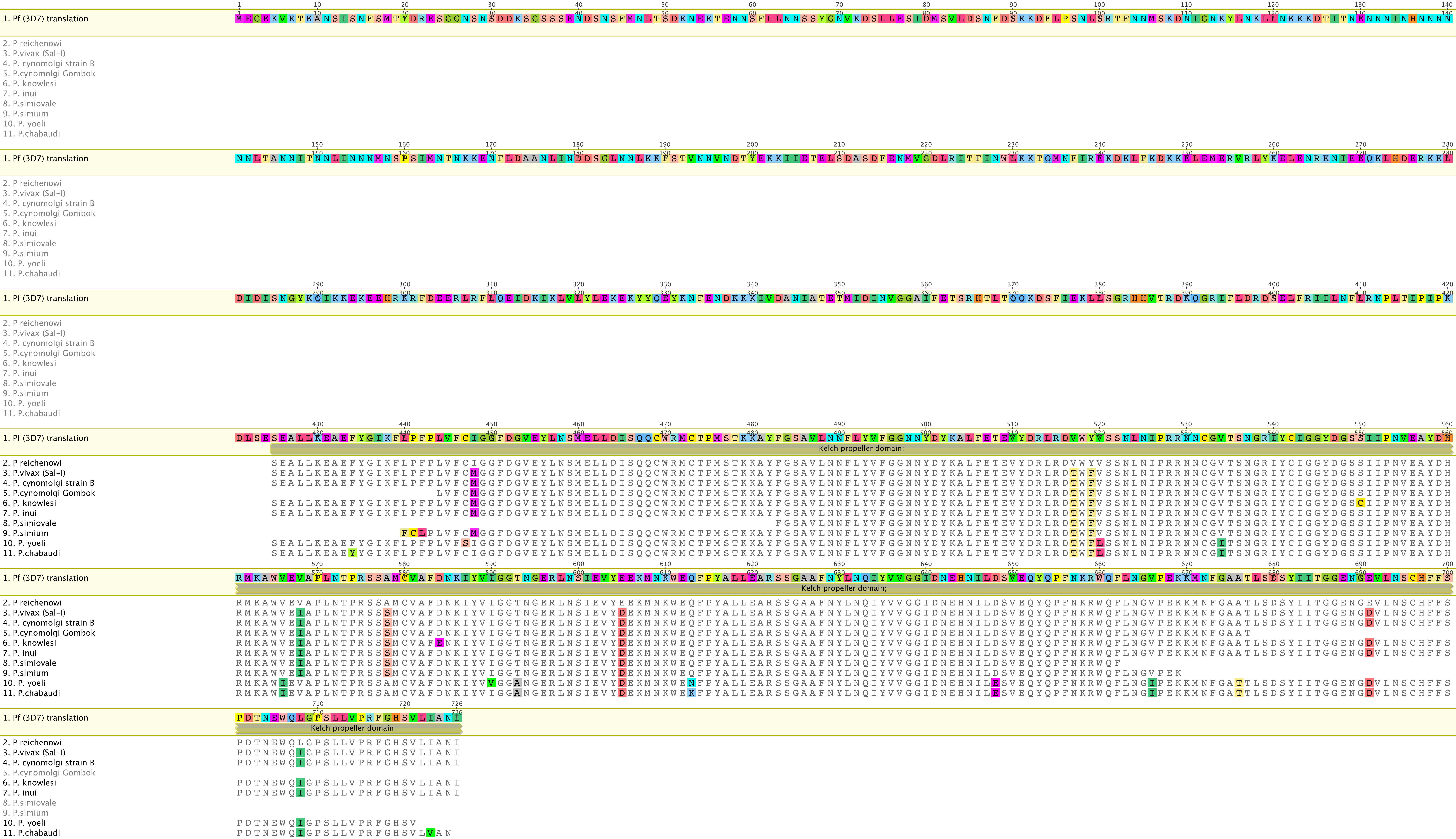
1. Pf (3D7) translation
2. P reichenowi
3. P.vivax (Sal-I)
4. P. cynomolgi strain B
5. P.cynomolgi Gombok
6. P. knowlesi
7. P. inui
8. P.simiovale
9. P.simium
10. P. yoeli
11. P.chabaudi
Kelch propeller domain;
