# Supplementary figures and images for: Genetic Analysis and Species Specific Amplification of the Artemisinin Resistance-Associated Kelch Propeller Domain in P. falciparum and P. vivax
Source: PLoS One. 2015 Aug 20;10(8):e0136099. doi: 10.1371/journal.pone.0136099 (PMC4546394; doi:10.1371/journal.pone.0136099)

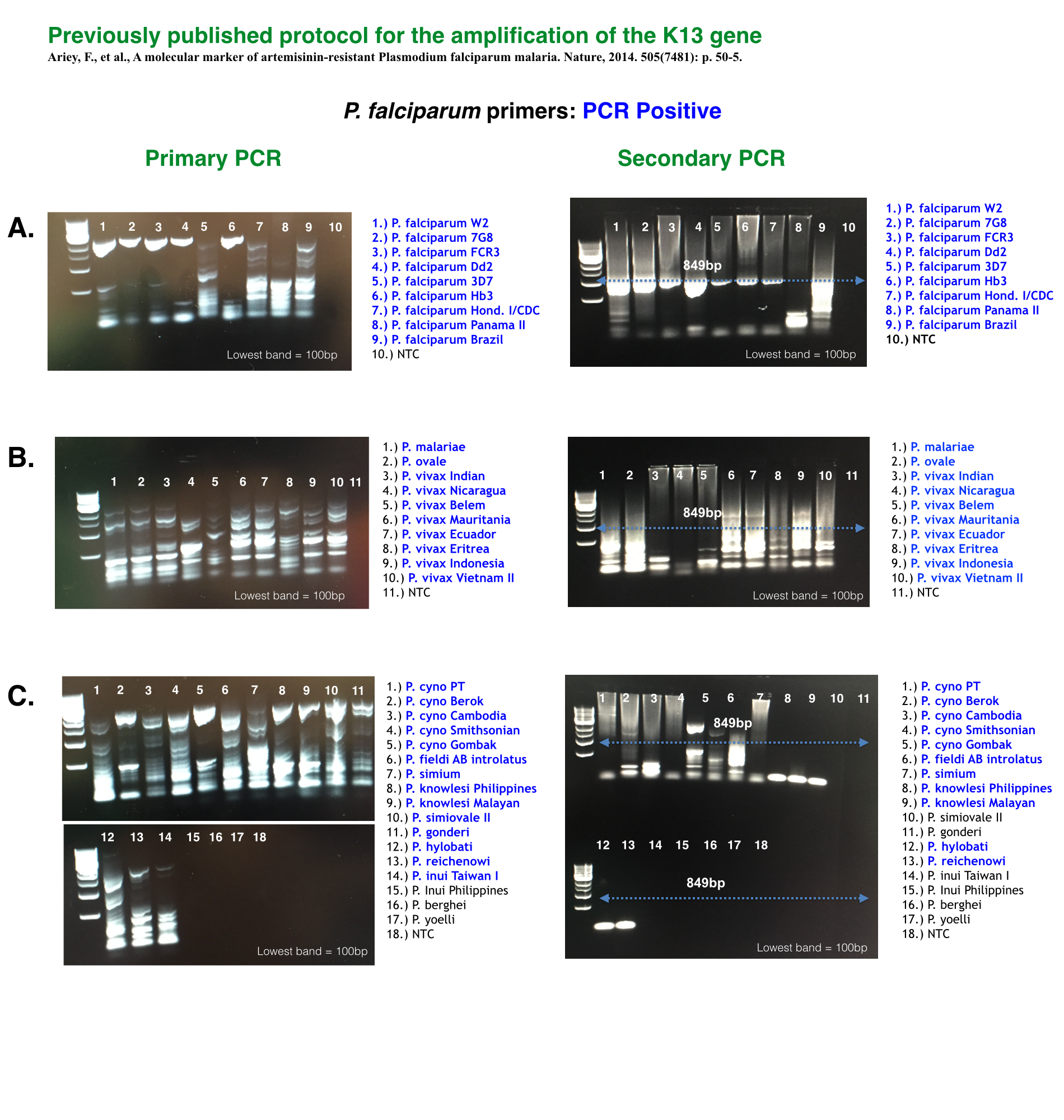

Supplement: S3 Fig — The original K13 gene amplification protocol was tested on multiple Plasmodium species, including human, rodent, and non-human primate malaria parasites from various geographical regions. Rows (A) and (B) show human malaria parasites tested; Row (C) shows non-human primate and rodent malaria parasites tested. Results are shown separately for the primary and secondary reactions. Blue text denotes samples that were amplified by the PCR protocol. (TIFF) [file pone.0136099.s003.tiff]
